# Determination of Laser-Induced Fluorescence Lifetimes Excited by Pulses of Comparable Duration

**DOI:** 10.1177/00037028251332975

**Published:** 2025-04-17

**Authors:** Lize Coetzee, Esa Jaatinen

**Affiliations:** Faculty of Science and Centre for Material Science, 1969 Queensland University of Technology, Brisbane, Australia

**Keywords:** Fluorescence spectroscopy, fluorescence lifetime, laser-induced fluorescence spectroscopy, LIF, stand-off sensors

## Abstract

This paper presents a novel analytical technique for evaluating fluorescence lifetimes excited by a nanosecond pulsed laser using a linearized rate equation approach that accounts for the incident pulse temporal distribution, an equivalent instrument response function, and non-exponential fluorescence decay which limits the application of traditional fluorescence lifetime techniques in stand-off applications. The approach is applied to model the fluorescence of a variety of pharmaceutical powders and phosphorescing samples exhibiting non-exponential decay and compared to results obtained with the maximum entropy method. Fluorescence lifetimes are found to be 3–5  ns, typical for organic fluorescent powders, and phosphorescence lifetimes were on the order of hundreds of nanoseconds. The approach also shows potential for determining the composition of mixed samples and can be readily extended to model increasingly complex scenarios with additional fluorescing or phosphorescing components.

## Graphical abstract

**Graphical abstract:**
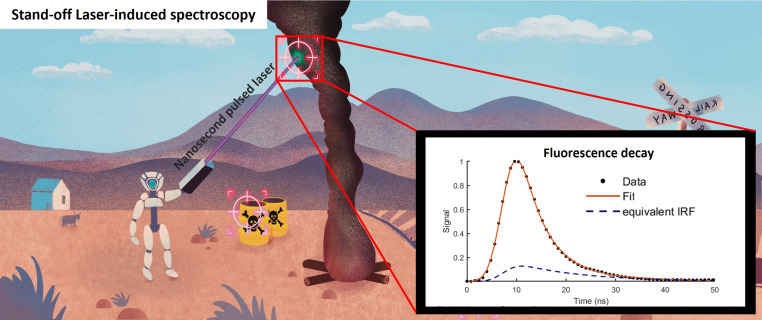
This is a visual representation of the abstract.

## Introduction

Laser-induced fluorescence (LIF) has been used since the advent of the laser in the 1960s to identify molecules through a measure of the number, wavelength, and time which photons are emitted after excitation. While it is a relatively mature diagnostic technique, over the last 10 years LIF has generated significant interest in security and bioterrorism applications^1–3^ such as aerosol monitoring^4–11^ and the identification of pharmaceutical products and drugs.^12–23^ It is also extensively used for food quality monitoring,^24,25^ the detection of crude oils,^26–28^ and biological materials in planetary exploration.^29^

Intrinsic or autofluorescence is a type of fluorescence which results from fluorophores often found in biological materials which fluoresce naturally without requiring the addition of fluorescent dyes or enhancers.^30^ Fluorescence occurs when a molecule is excited by incident light, with the relaxation process resulting in photon emission at a shifted wavelength. Materials and compounds can be identified by analyzing the spectral composition or temporal decay of fluorescence, or a combination of the two which has been shown to improve classification accuracy.^3^

Although the fluorescence lifetime of bulk materials such as clinical or pharmaceutical samples has often been determined by fitting a single exponential to the emitted fluorescence,^23,31–33^ it has also been shown that the lifetime of fluorophores such as tryptophan or samples with multiple fluorescing components are better determined using double or triple exponentials.^25,34–40^ However, studies as early as the 1980s have shown that although adding additional exponential terms can provide a more complete description each added term introduces a greater margin of error, and furthermore there exists samples which exhibit non-exponential decay.^34,35^ Some alternative strategies that have been used to model fluorescence lifetime include the use of stretched exponentials,^41^ limiting the measurement to capture decay from only short-lived fluorophores in a particular time range,^42^ or making use of computational analytical procedures which aim for identification rather than lifetime measurements.^43^ Furthermore, gated methods have seen some success,^44^ as well as convolving the profile of an excitation source for a laser with a theoretical model function and then comparing to the experimental data.^35–37,45^ The maximum entropy method (MEM) was developed to more accurately determine multiple lifetime components simultaneously when the number of decay components present are unknown.^46–49^ This is achieved by modelling the fluorescence decay as a weighted sum of exponentials and determining a weight distribution of lifetime components which best satisfies the observed data. However, as with other techniques, MEM can be sensitive to the initial parameters and initialization values. Furthermore, this method works best when experimental data contains discrete lifetime values rather than a continuous distribution.

A challenge with many fluorescence lifetime measurement techniques such as time correlated single photon counting (TCSPC)^38,39,50^ is the determination of an instrument response function (IRF) to a zero-lifetime sample. The IRF must be considered in signal analysis to ensure accurate fluorescence lifetime determination and avoid introducing systematic errors that may confuse material identification.^44,51^ Deconvolutional analysis which is commonly used to account for the IRF is only valid when the excitation pulse width is much shorter than the fastest measured decay time.^44^ The use of pico- or femto-second lasers can make IRF deconvolution more practical, however such laser sources are expensive and impractical for field use or when stand-off detection is required. Also, current deconvolution methods are numerical techniques, which can limit accuracy and can make it difficult to correlate results with underlying environmental conditions such as sample composition and age.^51^

For high accuracy laboratory-based measurement of fluorescence, photomultiplier tubes (PMTs) or single photon avalanche diodes (SPADs) are employed due to their high sensitivity. However, SPADs are not practical for stand-off applications as they are easily saturated.^52^ PMTs have excellent detection efficiency in the ultraviolet (UV) region, however this decreases in the visible and infrared regions.^53^ Thus, for a sensor which aims to identify materials over a broad range of wavelengths, compact avalanche photodiodes (APDs) that can operate under ambient conditions in the field are often used.^54,55^ However, a drawback with APDs is that they have a wavelength dependent temporal response due to the diffusion of carriers below the active junction which results in a wavelength dependent exponential tail in the IRF.^44,45,55^ While this can be corrected to some degree by approximating the IRF using the temporal profile of a scattered laser pulse, or calibration using a fluorophore with a known lifetime, systematic errors can be difficult to remove completely.^45,55^

In this paper we present an analytical model based on linearized population rate equations that describes the temporal evolution of sample fluorescence from the time that the laser pulse first interacts with the sample. This allows accurate determination of fluorescence lifetimes including for samples that exhibit non-exponential fluorescence decay. This approach conveniently accounts for the wavelength dependent IRF of the APD making it suitable in stand-off LIF devices to identify materials where convolution or deconvolution methods are impractical. We demonstrate the effectiveness of the method by modelling the fluorescence decay of various pharmaceutical powders and phosphorescent samples which demonstrate non-exponential decay when excited by nanosecond pulsed light. Our model also has the potential to allow the composition of mixed samples in some situations to be determined and can be expanded to reveal lifetimes when multiple fluorophores or phosphorescence is present. Our technique provides a practical approach to fluorescence lifetime determination for stand-off material identification which can be utilized in field settings.

## Theoretical Framework

**Figure 1.**
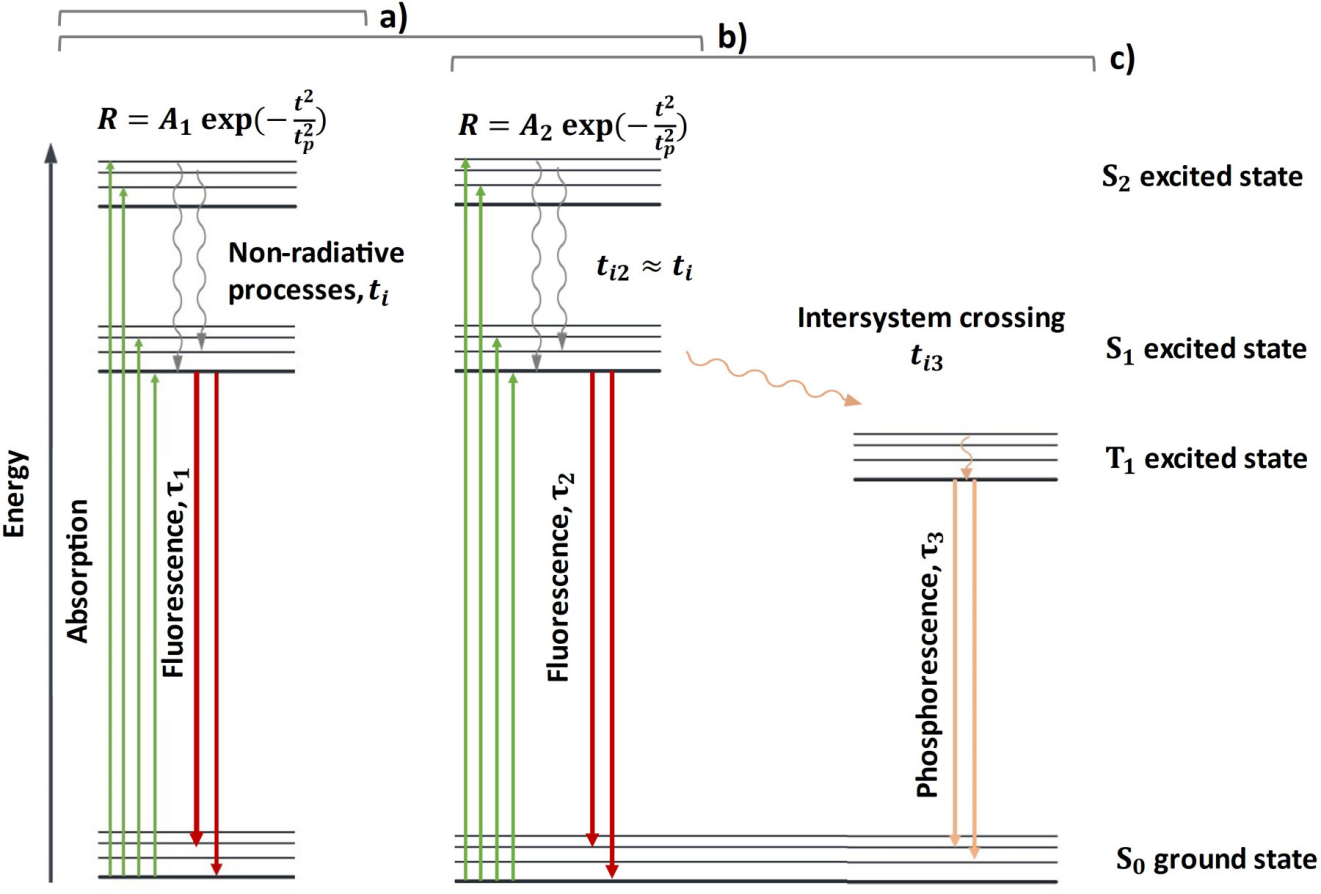
Energy diagram showing a molecule containing (a) a single fluorescing component, (b) multiple fluorescing components, and (c) demonstrating phosphorescence.

Fluorescence lifetime is a measure of how long a fluorophore emits light after excitation and offers a means to differentiate and identify materials. Electron pairs in the ground singlet state (S_0_) with opposite spins can be excited to an excited singlet state (S_1_). In some cases, an excited triplet state (T_1_) is also possible where the molecule must undergo intersystem crossing (ISC) to produce unpaired electrons with the same spin, although this process is relatively rare as this transition is symmetry forbidden. Emission from other excited states (S_2_) is also possible though less common in biological molecules.^44^ These fluorescence and phosphorescence pathways are shown in Figure 1.

There is some contradicting evidence regarding typical decay factors and how best to model them. Fluorescence is inherently multiexponential as substances generally contain a combination of fluorophores which each have distinct lifetimes contributing fractional amounts to the total lifetime.^30^ A typical model which can be used to broadly characterize molecules as organic or inorganic is given by Eq. 1.

(1) $$I(t)=I_{0}\exp\left(-\frac{t}{\underline{\tau }\;}\right)$$

where I(t) is the signal intensity at some time *t*, $I_{0}$ is the initial intensity, and $\underline{\tau }\;$ is the decay factor. Typically, inorganic molecules have decay factors on the order of micro- or milliseconds, while organic molecules have lifetimes below 100 ns.^33^

In the case where multiple, *n*, fluorophores may be present, Eq. 1 can be rewritten with individual lifetimes $\tau_{i}\;$ where each exponential component contributes some fraction $F_{i}$ as described by Eq. 2.

(2) $$I(t)=\sum_{i=1}^{n}\;F_{i}\exp\left(-\frac{t}{\tau_{i}}\right)$$

If a nanosecond pulsed laser is used as the excitation source, fluorescence from the sample will have commenced while the pulse is still pumping more of the population into the upper state. This impacts the temporal distribution of the emitted fluorescence which will not be strictly exponential and depend on the incident pulse duration and temporal distribution, often assumed to be Gaussian.

In principle, the fluorescence lifetime could be extracted by determining the IRF and numerically deconvolving it with the raw data or equivalent. However as discussed, these approaches have practical limitations for both stand-off measurements undertaken in the field and lab-scale measurements, arising from noise in the detection scheme and distorted signals. Hence, here we present a model which does not utilize deconvolution and accounts for the temporal distribution of the laser pulse and the APD IRF and can be applied to various fluorescing and phosphorescing systems.

### Single Fluorescing Systems

Figure 1 demonstrates some of the relaxation pathways after a population has been optically pumped at a rate *R* into an upper state. In the simplest case, when a single fluorophore is present without an accessible triplet state, the number of molecules entering an excited state is proportional to the laser pumping rate equation derived from a Gaussian temporal pulse, as given by Eq. 3.

(3) $$R(t)=A_{1}\exp\left(-\frac{{\left(t-t_{i}\right)}^{2}}{t_{p}^{2}}\right)$$

where $A_{1}$ is analogous to the quantum yield of the fluorophore, $t_{i}$ is a peak offset value, and $t_{p}$ is the pulse half width.

For a single fluorescing system as shown in Figure 1a, the pumped population will decay via fast non-radiative decay to the lowest excited level of the fluorescence band $S_{1}$ , with concentration $\left|S_{1}\right|$ , over a time $t_{i}$ which is typically much shorter than the duration of the laser pulse.^13^ This population will then radiatively decay via fluorescence to the ground state band with a lifetime $\tau_{1}$ . The temporal change in the $S_{1}$ population is described by a linearized rate equation used to describe optically pumped processes,^56,57^ as given by Eq. 4.

(4) $$\frac{d|S_{1}|}{dt}=R-\frac{\left|S_{1}\right|}{\tau_{1}}$$

Solving Eq. 4 and re-writing in terms of fluorescence signal intensity I(t) at some time *t* models the resulting fluorescence signal from a single fluorophore where $t_{i}$ is the non-radiative relaxation time offset, $t_{p}$ is the laser pulse half width, and $\tau_{1}$ is the fluorescence lifetime of the fluorophore.

(5) $$I(t)=A_{1}\exp\;\left(-\frac{t}{\tau_{1}}+\frac{4\tau_{1}t_{i}+t_{p}^{2}}{4\tau_{1}^{2}}\right)\left(\mathrm{erf}\left(\frac{t}{t_{p}}-\frac{t_{i}}{t_{p}}-\frac{t_{p}}{2\tau_{1}}\right)+\mathrm{erf}\left(\frac{t_{i}}{t_{p}}+\frac{t_{p}}{2\tau_{1}}\right)\right)$$

where the error function, erf, results from a finite integration of a Gaussian: $\mathrm{erf}(a)=\frac{2}{\sqrt{\pi }}\int_{0}^{a}\;\exp[-x^{2}]dx$ .

Equation 5 can be further adjusted to incorporate the APD response through the inclusion of a second exponential component as given by Eq. 6. This approximates the average effect of multiple wavelengths interacting with the APD due to the sample fluorescence, where it is known that the wavelength dependent tail for an APD tends to be exponential for each wavelength.^44^

(6) $$I(t)=A_{1}\exp\;\left(-\frac{t}{\tau_{1}}+\frac{4\tau_{1}t_{i}+t_{p}^{2}}{4\tau_{1}^{2}}\right)\left(\mathrm{erf}\left(\frac{t}{t_{p}}-\frac{t_{i}}{t_{p}}-\frac{t_{p}}{2\tau_{1}}\right)+\mathrm{erf}\left(\frac{t_{i}}{t_{p}}+\frac{t_{p}}{2\tau_{1}}\right)\right)+A_{\mathrm{APD}}\exp\left(-\frac{t}{\tau_{\mathrm{APD}}}+\frac{4\tau_{\mathrm{APD}}t_{i}+t_{p}^{2}}{4\tau_{\mathrm{APD}}^{2}}\right)\left(\mathrm{erf}\left(\frac{t}{t_{p}}-\frac{t_{i}}{t_{p}}-\frac{t_{p}}{2\tau_{\mathrm{APD}}}\right)+\mathrm{erf}\left(\frac{t_{i}}{t_{p}}+\frac{t_{p}}{2\tau_{\mathrm{APD}}}\right)\right)$$

where $A_{\mathrm{APD}}$ is a scaling factor for the signal contribution from the wavelength dependent tail, and $\tau_{\mathrm{APD}}$ is the average lifetime of the wavelength dependent tail.

### Multiple Fluorophores

This approach can be readily extended to model a material which contains multiple fluorophores. The fluorescence intensities of these fluorophores are independent of each other and occur simultaneously as separate processes. In this situation the linearized rate equations for the lower excited level of each of the two fluorescence bands are given by:

(7) $$\begin{array}{rl} & \frac{d|S_{1.1}|}{dt}=A_{1}\exp\left(-\frac{{\left(t-t_{i}\right)}^{2}}{t_{p}^{2}}\right)-\frac{\left|S_{1.1}\right|}{\tau_{1}}\\ & \frac{d|S_{1.2}|}{dt}=A_{2}\exp\left(-\frac{{\left(t-t_{i}\right)}^{2}}{t_{p}^{2}}\right)-\frac{\left|S_{1.2}\right|}{\tau_{2}} \end{array}$$

where $\tau_{1}$ and $\tau_{2}$ are the lifetimes of the two fluorophores as shown in Figure 1b. Note that the two offset times, *t_i_*, for the two fluorophores have essentially the same value due to the fast nature of nonradiative processes that are many orders of magnitude shorter than most fluorescence lifetimes.^30^ The scaling factors $A_{1}$ and $A_{2}$ depend on both the quantum yield and concentration of each fluorophore. These two equations can be solved and added to provide the intensity of the fluorescence signal, while also incorporating a third term to account for the APD response as given by Eq. 8.

(8) $$I(t)=A_{1}\exp\;\left(-\frac{t}{\tau_{1}}+\frac{4\tau_{1}t_{i}+t_{p}^{2}}{4\tau_{1}^{2}}\right)\left(\mathrm{erf}\left(\frac{t}{t_{p}}-\frac{t_{i}}{t_{p}}-\frac{t_{p}}{2\tau_{1}}\right)+\mathrm{erf}\left(\frac{t_{i}}{t_{p}}+\frac{t_{p}}{2\tau_{1}}\right)\right)+A_{2}\exp\;\left(-\frac{t}{\tau_{2}}+\frac{4\tau_{2}t_{i}+t_{p}^{2}}{4\tau_{2}^{2}}\right)\left(\mathrm{erf}\left(\frac{t}{t_{p}}-\frac{t_{i}}{t_{p}}-\frac{t_{p}}{2\tau_{2}}\right)+\mathrm{erf}\left(\frac{t_{i}}{t_{p}}+\frac{t_{p}}{2\tau_{2}}\right)\right)+A_{\mathrm{APD}}\exp\left(-\frac{t}{\tau_{\mathrm{APD}}}+\frac{4\tau_{\mathrm{APD}}t_{i}+t_{p}^{2}}{4\tau_{\mathrm{APD}}^{2}}\right)\left(\mathrm{erf}\left(\frac{t}{t_{p}}-\frac{t_{i}}{t_{p}}-\frac{t_{p}}{2\tau_{\mathrm{APD}}}\right)+\mathrm{erf}\left(\frac{t_{i}}{t_{p}}+\frac{t_{p}}{2\tau_{\mathrm{APD}}}\right)\right)$$

This can be further expanded to fit any number of fluorescing components, n, as seen in Eq. 9.

(9) $$\$\begin{array}{rl} & I(t)=\sum_{\;j=1}^{n}\left(A_{j}\exp\;\left(-\frac{t}{\tau_{j}}+\frac{4\tau_{j}t_{i}+t_{p}^{2}}{4\tau_{j}^{2}}\right)\left(\mathrm{erf}\left(\frac{t}{t_{p}}-\frac{t_{i}}{t_{p}}-\frac{t_{p}}{2\tau_{j}}\right)+\mathrm{erf}\left(\frac{t_{i}}{t_{p}}+\frac{t_{p}}{2\tau_{j}}\right)\right)\right)+\\ & A_{\mathrm{APD}}\exp\;\left(-\frac{t}{\tau_{\mathrm{APD}}}+\frac{4\tau_{\mathrm{APD}}t_{i}+t_{p}^{2}}{4\tau_{\mathrm{APD}}^{2}}\right)\left(\mathrm{erf}\left(\frac{t}{t_{p}}-\frac{t_{i}}{t_{p}}-\frac{t_{p}}{2\tau_{\mathrm{APD}}}\right)+\mathrm{erf}\left(\frac{t_{i}}{t_{p}}+\frac{t_{p}}{2\tau_{\mathrm{APD}}}\right)\right) \end{array}\$$$

where $A_{j}$ is analogous to the quantum yield of any fluorophore and $\tau_{j}$ is the corresponding lifetime.

### Fluorescing and Phosphorescing Systems

The approach can also be applied to the situation where ISC to a lower energy triplet state is available, and thus a molecule exhibits both fluorescence and phosphorescence. In this case, the rate equations describing the fluorescence and the phosphorescence can be written as

(10) $$\begin{array}{rl} & \frac{d|S_{1}|}{dt}=A_{2}\exp\;\left(-\frac{{\left(t-t_{i}\right)}^{2}}{t_{p}^{2}}\right)-\frac{\left|S_{1}\right|}{\tau_{2}}-\frac{\left|S_{1}\right|}{\tau_{23}}\\ & \frac{d|T_{1}|}{dt}=\frac{\left|S_{1}\right|}{\tau_{23}}-\frac{\left|T_{1}\right|}{\tau_{3}} \end{array}$$

where $\tau_{2}$ is the fluorescence lifetime, $\tau_{3}$ is the phosphorescence lifetime, and $\tau_{23}\;$ is the ISC rate of decay. Integration of Eq. 10 yields the intensity of the emission and is given by Eq. 11.

(11) $$\begin{array}{rl} & I(t)=A_{2}\exp\;\left(-\frac{t}{\tau_{2}}+\frac{4\tau_{2}t_{i}+t_{p}^{2}}{4\tau_{2}^{2}}\right)\left(\mathrm{erf}\left(\frac{t}{t_{p}}-\frac{t_{i}}{t_{p}}-\frac{t_{p}}{2\tau_{2}}\right)+\mathrm{erf}\left(\frac{t_{i}}{t_{p}}+\frac{t_{p}}{2\tau_{2}}\right)\right)+A_{3}\exp\;\left(-\frac{t-t_{i3}}{\tau_{3}}\right)\times \\ & \left(\exp\;\left(-\frac{4\tau_{2}t_{i}+t_{p}^{2}}{4\tau_{2}^{2}}\right)\left(\mathrm{erf}\left(\frac{t-t_{i}-t_{i3}}{t_{p}}\right)+\mathrm{erf}\left(\frac{t_{i}}{t_{p}}\right)\right)-\exp\;\left(-\frac{t-t_{i3}}{\tau_{2}}\right)\left(\mathrm{erf}\left(\frac{t-t_{i}-t_{i3}}{t_{p}}-\frac{t_{p}}{2\tau_{2}}\right)+\mathrm{erf}\left(\frac{t_{i}}{t_{p}}+\frac{t_{p}}{2\tau_{2}}\right)\right)\right)\\ & \;\times \exp\;\left(\frac{4\tau_{2}t_{i}+t_{p}^{2}}{4\tau_{2}^{2}}\right)+A_{\mathrm{APD}}\exp\;\left(-\frac{t}{\tau_{\mathrm{APD}}}+\frac{4\tau_{\mathrm{APD}}t_{i}+t_{p}^{2}}{4\tau_{\mathrm{APD}}^{2}}\right)\left(\mathrm{erf}\left(\frac{t}{t_{p}}-\frac{t_{i}}{t_{p}}-\frac{t_{p}}{2\tau_{\mathrm{APD}}}\right)+\mathrm{erf}\left(\frac{t_{i}}{t_{p}}+\frac{t_{p}}{2\tau_{\mathrm{APD}}}\right)\right) \end{array}$$

where $A_{2}$ is the fluorescence quantum efficiency and $A_{3}$ is the phosphorescence quantum efficiency.

### Determining Lifetimes Using Least Squares Fitting and Maximum Entropy Method

Least squares fitting can be used with Eqs. 6, 9, and 11 to determine suitable values for lifetimes and other parameters which fit with the experimental data. This can be easily achieved using various software packages. However, fitting a large number of parameters may result in large uncertainties and results that are not physically meaningful. Furthermore, there could be multiple solutions that provide a good fit. Thus here, MEM was used to confirm results.

A detailed outline of the MEM method is described by Esposito et al.^46^ In brief, for each data point, *m*, in the time resolved fluorescence decay data a theoretical function $T(t_{m})$ is defined.

(12) $$T(t_{m})=\overset{N}{\underset{k=1}{\sum }}\;C_{m,k}\cdot \alpha_{k}$$

$C_{m,k}$ describes the theoretical decay of the sample and is traditionally a convolution of the IRF with an assumed sum of exponential decay components. Applying it to the approach used here by substitution of Eq. 5 yields:

(13) $$\begin{array}{r} C_{m,k}=\overset{M}{\underset{m=1}{\sum }}\;{\left(\exp\;\left(-\frac{t_{m}}{\tau_{k}}+\frac{4\tau_{k}t_{i}+t_{p}^{2}}{4\tau_{k}^{2}}\right)\left(\mathrm{erf}\left(\frac{t_{m}}{t_{p}}-\frac{t_{i}}{t_{p}}-\frac{t_{p}}{2\tau_{k}}\right)+\mathrm{erf}\left(\frac{t_{i}}{t_{p}}+\frac{t_{p}}{2\tau_{k}}\right)\right)\right)}_{\;} \end{array}$$

The MEM method then aims to select a distribution of lifetimes and weights which maximizes the Skilling entropy function, as outlined by Esposito et al.^46^

## Experimental

### Materials and Methods

**Figure 2.**
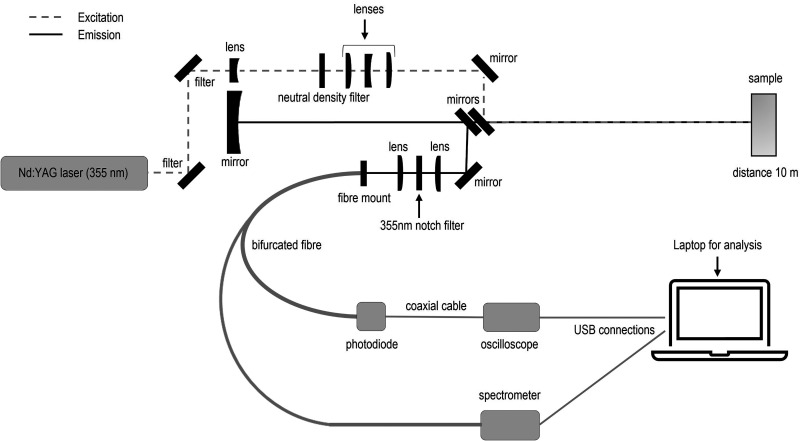
The experimental setup used to obtain fluorescence spectra and lifetime decay profiles. It includes a dedicated optical unit for stand-off spectroscopy that combines light delivery and capture.

A schematic of the experimental setup used is shown in Figure 2. Fluorescence data was obtained using a nanosecond pulsed laser (Quantel Ultra, 355  nm, 20  Hz, 4  ns pulse duration, ∼ 2 mJ/pulse, interpulse energy variability ∼ 10%) at a stand-off distance of 10  m. The temporal fluorescence decay was measured with an A-Cube series APD (Laser Components, A-Cube S500-240) coupled to a high speed PicoScope (Pico Technology, PicoScope 6402, 350  MHz, 5 GS/s). Analysis of temporal decay curves were performed using the curve fitting tool on Matlab, which utilized least-squares regression. The temporal profile of the laser was measured to ensure consistency and accuracy for fitted parameters such as $t_{p}$ and $t_{i}$ , and to validate the assumption that the temporal pulse profile is Gaussian in nature.

In this investigation the temporal fluorescence decay signal captured was the sample emission across the entire 200  nm to 900  nm spectral range of the APD. To demonstrate that the approach presented here can negate the impact of the wavelength dependent IRF regardless of the spectral composition of a material’s fluorescence, the collected signal was split using a bifurcated fiber to simultaneously capture the temporal fluorescence decay and the fluorescence spectra resolved with a QE Pro (Ocean Optics) spectrometer. For very precise determination of individual fluorophore lifetimes in a laboratory setting it may be useful to make use of filters to capture the decay for a smaller region of wavelengths which can be more readily contributed to a single fluorophore. However, this requires prior knowledge of the material being investigated. While this is possible in laboratory settings where the aim is to measure and perhaps quantify a specific sample, here no spectral filters were used so as not to limit the range of materials that can be identified in stand-off mode.

The fluorescence decay for a variety of commercially available pharmaceutical samples were modelled using an average of 1000–2000 laser pulses. These included a pure aspirin sample, two types of coated aspirin (duentric and enteric), paracetamol, rash powder with maize starch, and a talcum powder. A summary of their excipients can be found in Table I. These were all prepared in powder form and placed in low-fluorescence quartz cuvettes. Commercially obtained phosphorescing water-based textile ink containing pigmented polymers, commercial zinc sulphide powder with dopants, and vinyl plastic containing phosphors were also used to model phosphorescence and non-exponential decay.

**Table I.** Summary of pharmaceutical samples and their lifetimes.

Name	Ingredients	Lifetime (ns)
Aspirin	Acetylsalicylic acid (ASA), maize starch	(5.2±0.6)
Duentric	ASA, purified talc, Sodium bicarbonate, guar gum, colloidal anhydrous silica, glycerol, hypromellose, macrogol 3350, methacrylic acid copolymer, polyvinyl alcohol, sodium lauryl sulfate, sunset yellow FCF aluminium lake, titanium dioxide, triethyl citrate	(4.2±0.3)
Enteric	ASA, maize starch, pregelatinised maize starch, citric acid, purified talc, Betadex, carbomer 941, hypromellose, lecithin, macrogol 6000, methacrylic acid copolymer, microcrystalline cellulose, polyvinyl alcohol, purified water, sodium hydroxide, stearic acid, sunset yellow FCF aluminium lake, titanium dioxide, xanthan gum	(4.8±0.5)
Paracetamol	Acetaminophen, maize starch, pregelatinised maize starch, purified talc, povidone, stearic acid, potassium sorbate	(5.1±0.6)
Rash powder	Maize starch, zinc oxide, calcium phosphate	(3.3±0.6)
Talc	Talcum powder, lavender extract, possible other excipients	(4.0±0.4)

## Results and Discussion

Table I shows the lifetimes for the bulk pharmaceutical powders determined from experimental data using least-squares fitting, which are within the range expected for similar samples.

**Figure 3.**
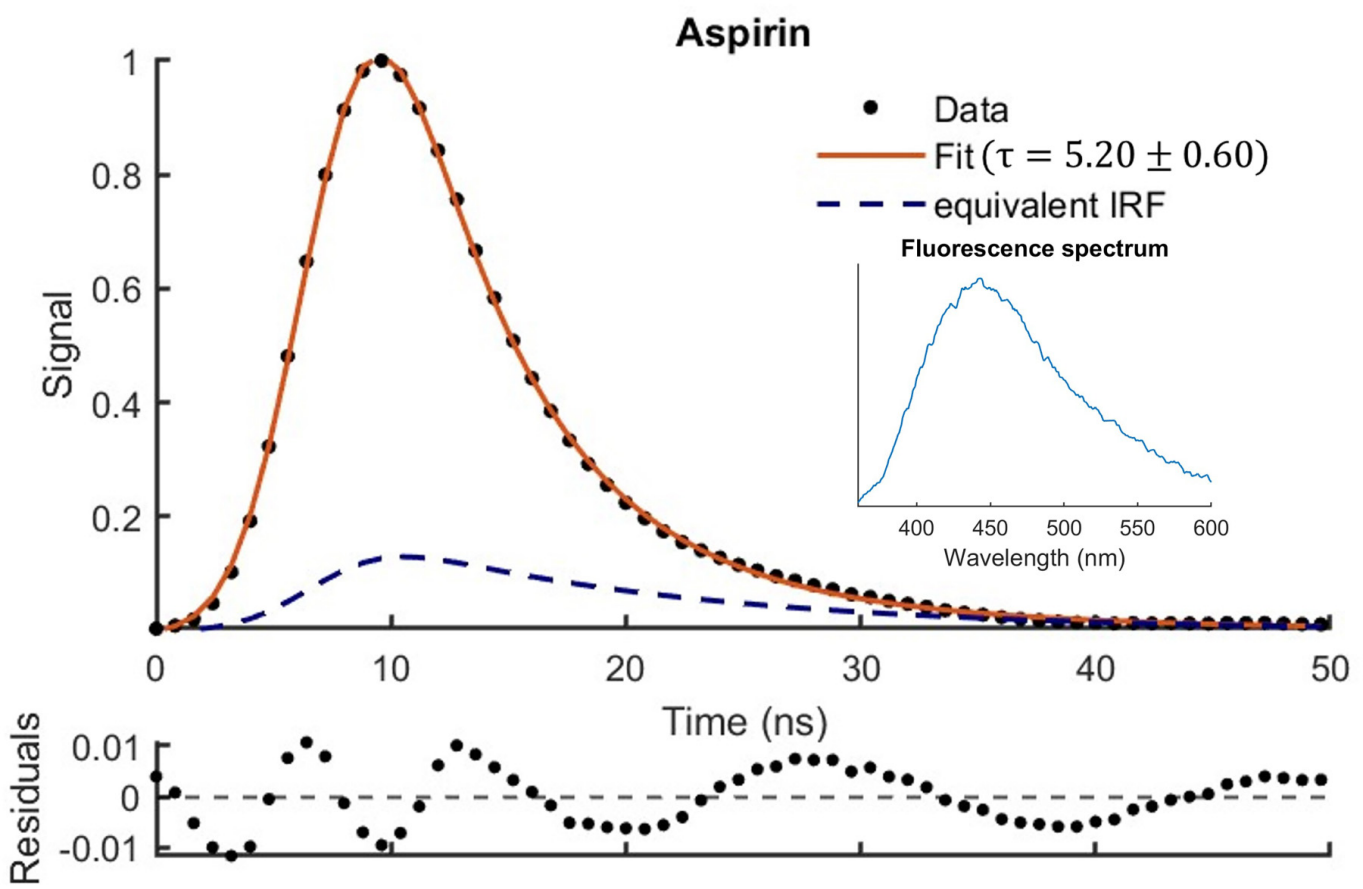
Fluorescence decay for the aspirin sample modelled using Eq. 6. The fluorescence spectrum is also shown.

Figure 3 shows the temporal variation of the fluorescence excited at 355  nm predicted by Eq. 6 for the aspirin sample using least squares fitting. The oscillations observed in the residuals have been previously reported when using APDs and are the result of high signal and APD gain^54^ but since their net average is zero they do not introduce systematic errors to the fit. Previously it has been shown that a visual inspection of the fit and residuals often provides a good determination on the quality of the fit to given data^44^ in situations where it is not possible to use the $\chi^{2}$ statistic typically used in TCSPC. The equivalent IRF shown in Figure 3 is described by the second component (APD) in Eq. 6 and corresponds to the APD response to the fluorescence and scatter generated by the laser pulse. In general, it was observed that the lifetime $\tau_{\mathrm{APD}}$ related to the wavelength dependent tail generally increased for samples that fluoresce at longer wavelengths as will be discussed. The calculated fluorescence lifetime value of (5.20±0.60)ns is well within the expected range for aspirin samples, when excited by UV light. The quality of the fit is good since the residuals do not indicate any systematic errors and increasing the sampling rate over 1 kS/s showed no noticeable improvement to the margin of error.

Equation 6 can also be used to fit the fluorescence decay of samples with more complex compositions, such as the enteric and paracetamol samples with the results given in Table I. Paracetamol, with acetaminophen as the active ingredient, had a lifetime of (5.10±0.60)ns . The lifetime of pure solid phase acetaminophen has previously been reported as (1.77±0.02)ns when excited at $\lambda_{\mathrm{em}}=382\;$ nm^23^ and 3.7 ns at $\lambda_{\mathrm{em}}=330$ nm and 1.9 ns at $\lambda_{\mathrm{em}}=425$ nm for aqueous acetaminophen.^58^ This demonstrates the impact that excipients and sample environment have on the fluorescence decay.

**Figure 4.**
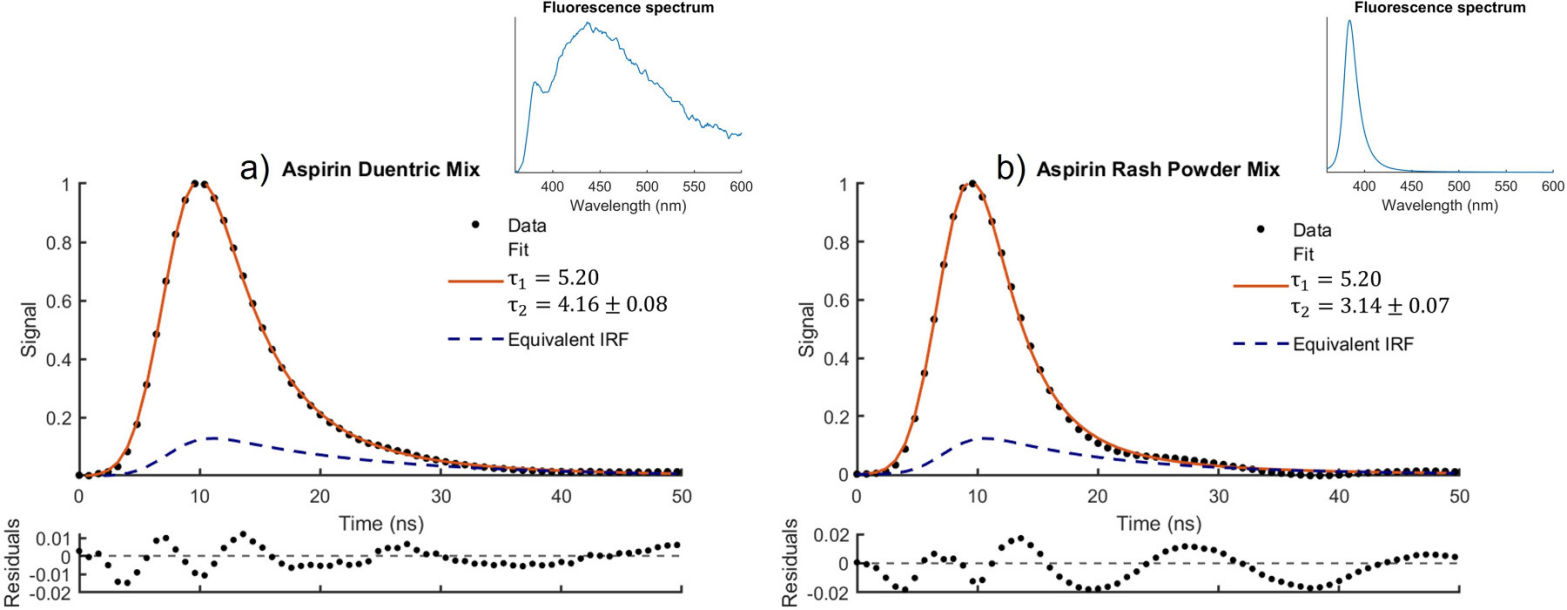
Fluorescence spectra and decay for mixtures of (a) aspirin and duentric and (b) aspirin and rash powder modelled using Eq. 9.

Equation 9 was used to model the decay of mixed fluorescing samples. This proved somewhat difficult if all parameters are unconstrained as the complexity of the model results in several possible solutions, and furthermore previous studies have shown difficulty in constraining fit parameters for complex models such as triple exponentials.^25,50^ The number of possible solutions were greatly reduced by constraining the parameters $t_{i},\;t_{p},\;A_{\mathrm{APD}},\;\tau_{\mathrm{APD}}$ to their measured values. However, there was still a tendency for the model to converge on solutions where $\tau_{1}$ and $\tau_{2}$ were the same in some cases. Thus, mixtures were treated with one known and unknown component, where the known component’s lifetime $\tau_{1}$ was also set as a fixed parameter. Figure 4 shows the result of this approach for two mixtures containing aspirin as the known and either duentric or rash powder as the unknown material. The calculated lifetime of (4.16±0.08) ns for duentric in the mixture agrees with the calculated lifetime of a pure duentric sample of (4.2±0.02) ns as shown in Table I. Similarly for rash powder, the lifetime calculated for the mixture was (3.14±0.07) ns compared the lifetime of a pure sample of (3.3±0.6) ns as shown in Table I. This shows that this model can provide information about the composition of the sample from the fluorescence lifetimes alone. This is the case even though the two mixed samples had very different fluorescence spectra, resulting in different IRFs for each sample.

MEM was used to verify the results obtained through least-squares fitting. Using this method for the aspirin sample, the lifetime probability density value peaked at ∼5.2 ns, which is in good agreement with the value obtained through the approach presented here as shown in Table I and Figure 3. Similar lifetimes were also found for the other unmixed samples. It should be noted that the lifetime distributions found using MEM were relatively broad and did not produce a second lifetime value to account for the decay from the APD tail, likely arising from its continuous nature. Furthermore, due to the collection of emissions from a broad range of wavelengths (whereas MEM is typically used for specific emission wavelengths or ranges with discrete distributions), there was a higher level of uncertainty in the acquired lifetimes.

Using MEM did not prove successful for separating the mixtures, once again likely due to the continuous nature of the lifetime distribution and the broad range of wavelengths over which the emission was captured, as well as the short separation between the expected lifetimes for the mixed components. However, the maximum probability density for each of the mixtures ranged from 4–6  ns. This is slightly longer than the lifetimes obtained using Eq. 9, where the artificial lengthening likely comes from the APD wavelength dependent tail. A sample of olive oil was found to fluoresce at both 355  nm and 532  nm with differing lifetimes. By exciting the sample with both wavelengths simultaneously we were able to use MEM to obtain two lifetimes of approximately 3.2  ns and 9.8  ns. This shows that under some circumstances MEM can be used to separate lifetime components over a broad range of wavelengths.

**Figure 5.**
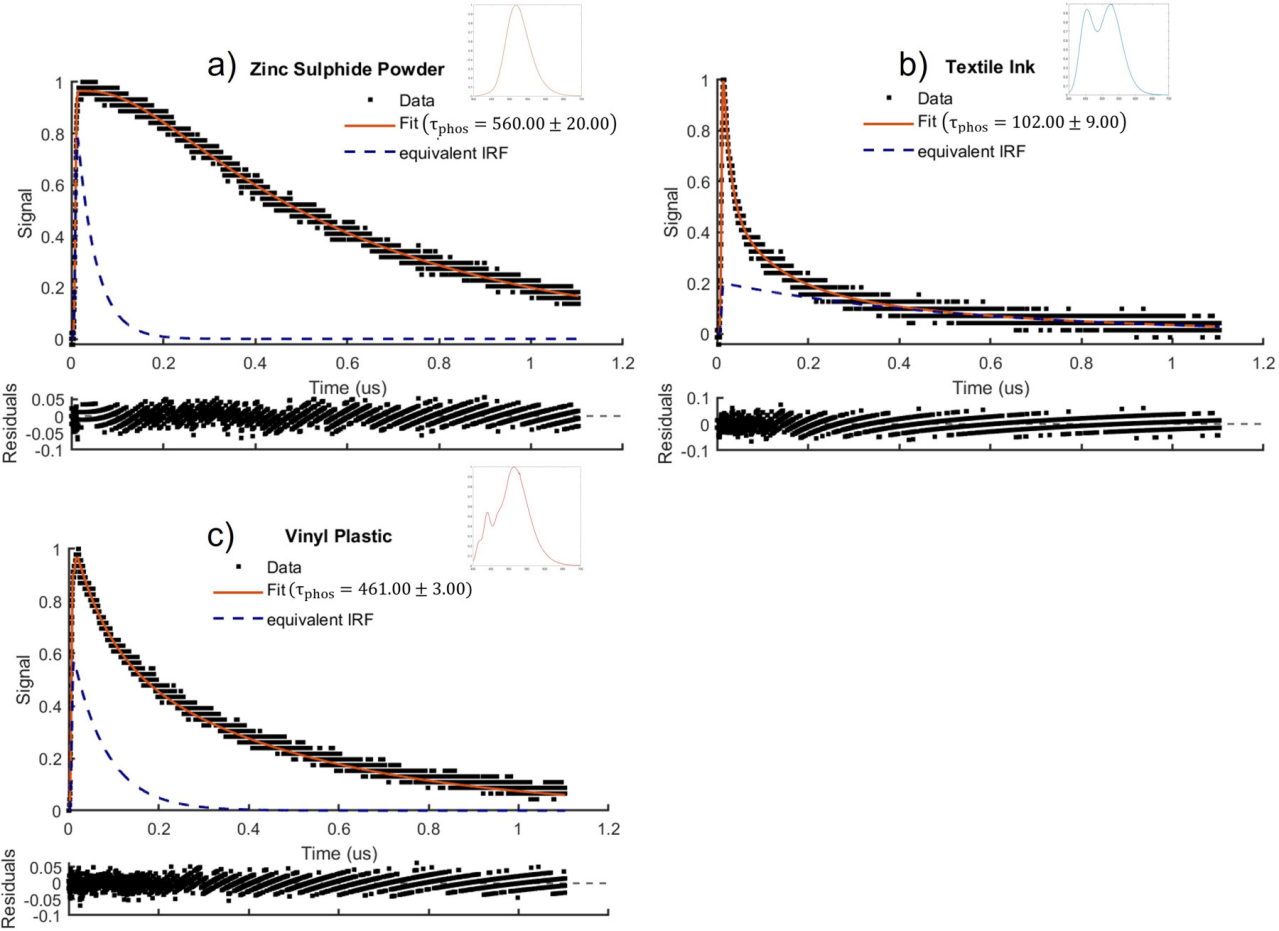
Phosphorescence decay modelled by Eq. 11 and fluorescence spectra for (a) zinc sulphide powder, (b) textile ink, (c) vinyl plastic.

The spectra and temporal decay of simultaneously fluorescing and phosphorescing samples of water-based textile ink containing pigmented polymers, commercial zinc sulphide powder with dopants, and vinyl plastic containing phosphors are shown in Figure 5. Eq. 11 was used to fit to the combined fluorescence and phosphorescence of each of these samples. The phosphorescence and fluorescence lifetimes were found to be (560±20) ns and (130±40) ns for the zinc sulphide powder, (102±9) ns and (15±6) ns for the textile ink, and (461±3) ns and (2.0±0.7) ns for the vinyl plastic.

**Figure 6.**
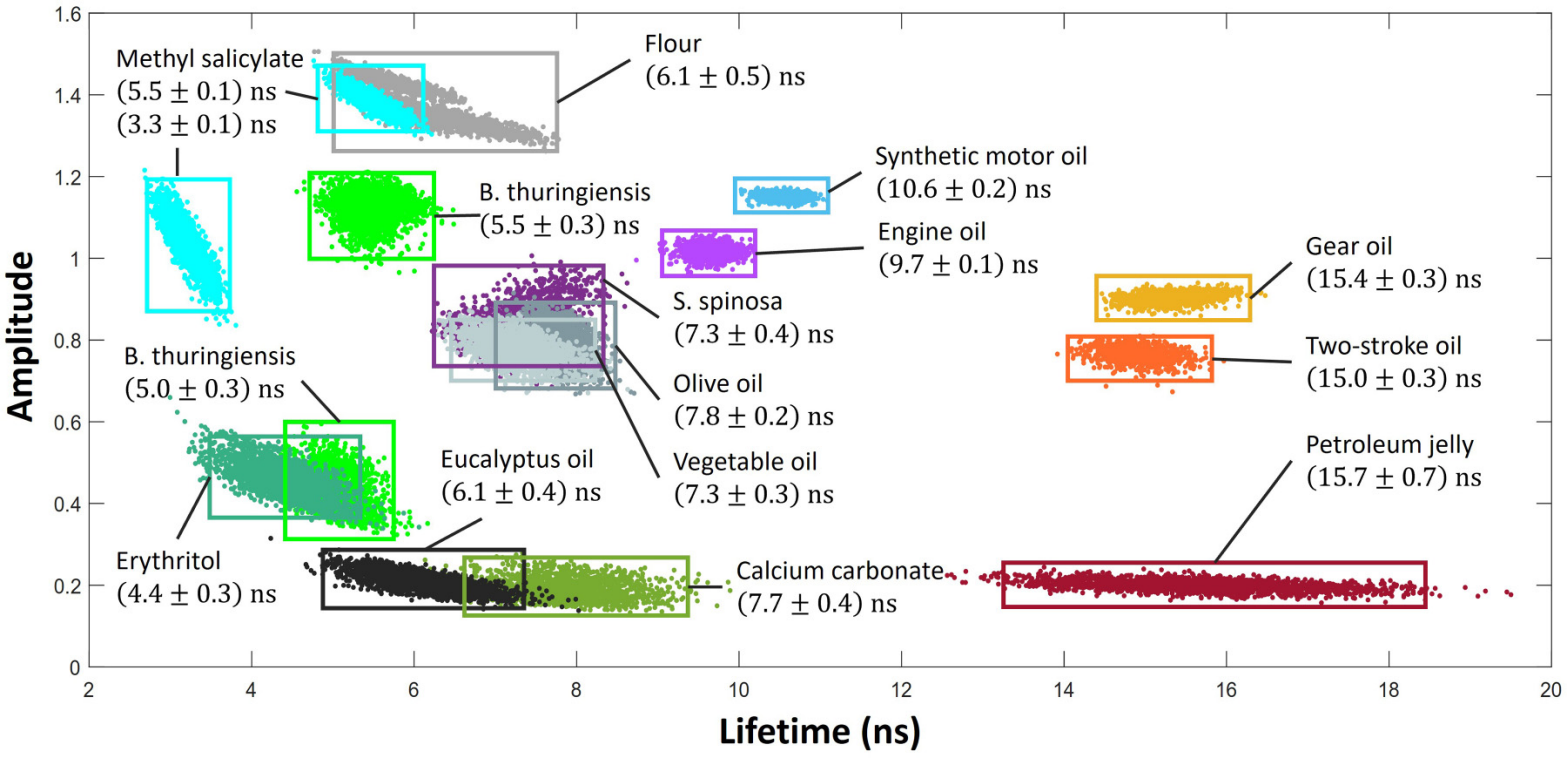
A selection of samples treated as bulk material with fluorescence decay curves modelled using Eq. 6 with least squares fitting, with the quoted lifetimes calculated from an average of 2000 pulses.

Equation 6 was used to fit to a variety of bulk samples using each individual pulse to observe the spread of calculated lifetimes. This can be seen in Figure 6, where the signal has not been normalized. An equal volume of each sample was used, and as such the amplitude values $A_{1}$ can be used as a comparison of their relative quantum efficiency. This is useful as in some cases the lifetimes of different samples overlap. However, care must be taken as it is not clear whether the samples are experiencing self-absorption which can lower their overall fluorescence signal. Two of the samples, *Bacillus thuringiensis* and methyl salicylate, were tested on two separate days and their lifetimes and intensities varied. For *B. thuringiensis* the lifetime decreased slightly from (5.5±0.3) ns to (5.0±0.3) ns, and for methyl salicylate it decreased significantly from (5.5±0.1) ns to (3.3±0.1) ns. This change is thought to be due to photochemical effects which may affect the fluorescence quantum yield, or simply sample ageing. It is unlikely to be a result of laser induced thermal degradation, since with an illuminated laser spot 10 mm in diameter and a maximum average laser power per unit area of 300 W/m2, the maximum possible temperature increase was calculated to be less than 1 °C, assuming that all light energy is absorbed by the sample. However, since the light absorption of methyl salicylate at 355 nm is very low,^59^ any actual sample temperature change would be substantially lower. In general, samples with a lower amplitude (corresponding to a lower fluorescence quantum efficiency) tend to have a larger spread in fluorescence lifetimes between pulses. This can be attributed to the high inter-pulse variability observed in the laser, measured to have a coefficient of variation (standard deviation as a percentage of the mean) of ∼ 10% for both the pulse power and duration. Furthermore, Q-switch jitter may result in lower energy pulses which have a broader width giving a larger distribution of lifetimes. It can also be seen that samples derived from crude oils (synthetic motor oil, engine oil, gear oil, two-stroke oil, and petroleum jelly) tend to have significantly longer lifetimes. This trend is in line with previous studies where it has been found that samples containing proteins have much shorter lifetimes than samples derived from crude oils, such as diesel fuels.^60^ Furthermore, choice of excitation wavelength can greatly affect fluorescence lifetime, such as one study which found that diesel fuels have lifetimes around 10 ns with 280 nm excitation but 14–30 ns with 340 nm excitation.^60^

## Conclusion

We have shown that our presented analytical model can be used to fit to raw temporal fluorescence data to determine lifetime values τ for a variety of pharmaceutical powders and samples that consist of single and multiple fluorophores, even in the presence of phosphorescence. An advantage of this model is that as an analytical approach it allows the determination of the IRF of the detection system which accounts for the laser temporal pulse distribution and wavelength dependent tail temporal response that occurs in APDs. This is particularly useful for stand-off detection where compact nanosecond pulsed lasers and APDs are used due to the need for lightweight and inexpensive equipment which makes traditional photon counting and deconvolution techniques impractical.

In future work, we aim to use fluorescence lifetime determination with an APD as a supplementary method to spectrometers for sample classification and identification in situations where fluorescence spectra are difficult to interpret, and additional information is required to aid in identification, without significantly increasing the footprint of the sensor. There is also potential to use this as an alternative, stand-alone technique for situations where the size, weight, and cost of the sensor is of utmost importance. Furthermore, using this set-up as a stand-alone technique is beneficial as it consists of a simple circuit attached to a diode which does not require specialized software to interpret spectra. This will increase the deployability and robustness of stand-off LIF sensors that are used to identify possible hazards in the field.

## References

[1] Buteau S. . “Spectral Laser Induced Fluorescence for Standoff Detection and Classification of Aerosolized Biological Threats”. Paper presented at: OSA Optical Sensors and Sensing Congress. Washington, DC; 19–23 July 2021.

[2] Mann M. Kumar S. Rao A.S. Sharma R.C. . “Remote Sensing for the Detection of Bio- and Non-Bioaerosols for Defence Applications”. Curr. Sci. 2020. 118(12): 1980–1983.

[3] Fellner L. Kraus M. Gebert F. Walter A. Duschek F. . “Multispectral LIF-Based Standoff Detection System for the Classification of CBE Hazards by Spectral and Temporal Features”. Sensors. 2020. 20(9): 2524. 32365598 10.3390/s20092524 PMC7249005

[4] Wang Y. Huang Z. Zhou T. Bi J. Shi J. . “Identification of Fluorescent Aerosol Observed by a Spectroscopic Lidar over Northwest China”. Opt. Express. 2023. 31(13): 22157. 37381296 10.1364/OE.493557

[5] Gabbarini V. Rossi R. Ciparisse J.F. Malizia A. , et al. “Laser-Induced Fluorescence (LIF) as a Smart Method for Fast Environmental Virological Analyses: Validation on Picornaviruses”. Sci. Rep. 2019. 9(1): 12598. 31467322 10.1038/s41598-019-49005-3 PMC6715700

[6] Gabbarini V. Rossi R. Ciparisse J.F. Puleio A. , et al. “An Ultraviolet Laser-Induced Fluorescence (UV-LIF) System to Detect, Identify and Measure the Concentration of Biological Agents in the Environment: a Preliminary Study”. J. Instrum. 2019. 14(07): C07009.

[7] Chen S. Chen Y. Zhang Y. Guo P. , et al. “Dual-Channel Mobile Fluorescence Lidar System for Detection of Tryptophan”. Appl. Opt. 2020. 59(3): 607. 32225184 10.1364/AO.378442

[8] Shoshanim O. Baratz A. . “Daytime Measurements of Bioaerosol Simulants Using a Hyperspectral Laser-Induced Fluorescence LIDAR for Biosphere Research”. J. Environ. Chem. Eng. 2020. 8(5): 104392.

[9] Kumar S. Parmar A. Sharma R.C. . “Remote Sensing of Biochemicals in Aerosol Form Using Fluorescence Sensor for Defence and Security”. IEEE Sens. J. 2019. 19(23): 11129–11133.

[10] Owoicho O. Olwal C.O. Quaye O. . “Potential of Laser-Induced Fluorescence-Light Detection and Ranging for Future Stand-off Virus Surveillance”. Microb. Biotechnol. 2021. 14(1): 126–135. 33242369 10.1111/1751-7915.13698 PMC7753352

[11] Kaliszewski M. Wlodarski M. Bombalska A. Kwasny M. , et al. “The Application of Semiconductor Based UV Sources for the Detection and Classification of Biological Material”. Proc. SPIE. 2013. 8703: 870305.

[12] Miles C.I. Schenk G.H. . “Fluorescence of Acetylsalicylic Acid in Solution and its Measurement in Presence of Salicylic Acid”. Anal. Chem. 1970. 42(6): 656–659. 5431519 10.1021/ac60288a032

[13] Smith J. Loxley K. Sheridan P. Hamilton T.M. . “Analysis of Caffeine in Beverages Using aspirin as a Fluorescent Chemosensor”. J. Chem. Educ. 2016. 93(10): 1776–1780.

[14] Voinov Y.P. Gorelik V.S. Umarov M.F. Morozova S.V. . “Difference Fluorescence Spectroscopy of the Structure and Composition of Bioactive Preparations”. Bull. Lebedev Phys. Inst. 2011. 38(11): 323–327.

[15] Moreira A. Dias I. Neto G. Zagatto E. , et al. “Solid-Phase Spectrofluorimetric Determination of Acetylsalicylic Acid and Caffeine in Pharmaceutical Preparations Using Partial Least-Squares Multivariate Calibration”. Talanta. 2005. 67(1): 65–69. 18970138 10.1016/j.talanta.2005.02.004

[16] Navalon A. Blanc R. Del Olmo M. Vilchez J. . “Simultaneous Determination of Naproxen, Salicylic Acid and Acetylsalicylic Acid by Spectrofluorimetry Using Partial Least-Squares (PLS) Multivariate Calibration”. Talanta. 1999. 48(2): 469–475. 18967486

[17] Villari A. Micali N. Fresta M. Puglisi G. . “Spectrofluorimetry at Zero Angle: Determination of Salicylic Acid in an Acetylsalicylic Acid Pharmaceutical Formulation”. Analyst. 1994. 119(7): 1561. 7943744 10.1039/an9941901561

[18] Fidanza J. Aaron J.J. . “The Analysis of Pharmaceutical Preparations Using Solid-Surface Room Temperature Photochemical-Fluorescence”. J. Pharm. Biomed. Anal. 1987. 5(6): 619–623. 16867486 10.1016/0731-7085(87)80074-2

[19] Kazakis N.A. Tsirliganis N.C. Kitis G. . “Preliminary Thermoluminescence and Optically Stimulated Luminescence Investigation of Commercial Pharmaceutical Preparations Towards the Drug Sterilization Dosimetry”. Appl. Radiat. Isot. 2014. 91: 79–91. 24922552 10.1016/j.apradiso.2014.05.012

[20] Kazakis N.A. Tsirliganis N.C. Kitis G. . “Post-Sterilization Radiation Dosimetry of Commercial Pharmaceuticals Using Optically Stimulated Luminescence”. Radiat. Phys. Chem. 2018. 150: 111–119.

[21] Etminan A. Uzunovic A. Topcagic A. Zero S. , et al. “Quantification of Active Substances in Some Drugs Using by Derivative UV/Vis spectroscopy”. Proc. IFMBE. 2020. 73: 553–557.

[22] Wang J. Chen G. Zhu T. Gao S. , et al. “Identification of Tartrazine and Sunset Yellow by Fluorescence Spectroscopy Combined with Radial Basis Function Neural Network”. Chin. Opt. Lett. 2009. 7(11): 1058.

[23] Moreira A.B. Oliveira H.P.M. Atvars T.D.Z. Dias I.L.T. , et al. “Direct Determination of Paracetamol in Powdered Pharmaceutical Samples by Fluorescence Spectroscopy”. Anal. Chim. Acta. 2005. 539(1–2): 257–261.

[24] Sikorska E. Romaniuk A. Khmelinskii I. V. Herance R. , et al. “Characterization of Edible Oils Using Total Luminescence Spectroscopy”. J. Fluoresc. 2004. 14(1): 25–35. 15622857 10.1023/b:jofl.0000014656.75245.62

[25] Chen M. Ma W. Shi L. Lai Y. , et al. “Detection of Multiple-Heated Edible Oil Based on Fluorescence Spectroscopy and Lifetime Method”. J. Appl. Spectrosc. 2022. 89(1): 126–133.

[26] Li X. Chen Y. Li J. Jiang J. , et al. “Time-Resolved Fluorescence Spectroscopy of Oil Spill Detected by Ocean Lidar”. Proc. SPIE. 2016. 10155: 101550Q.

[27] Mu T. Chen S. Zhang Y. Guo P. , et al. “Motor Oil Classification Based on Time-Resolved Fluorescence”. PLoS One. 2014. 9(7): 2100555 10.1371/journal.pone.0100555 PMC4079594 24988439

[28] Falla Sotelo F. Araujo Pantoja P. . Lopez-Gejo J. Le Roux G.A.C. , et al. “Application of Fluorescence Spectroscopy for Spectral Discrimination of Crude Oil Samples”. Braz. J. Petro. Gas. 2008. 2(2): 63–71.

[29] Laurent B. Cousins C.R. Gunn M. Huntly C. , et al. “UV Luminescence Characterisation of Organics in Mars-Analogue Substrates”. Icarus. 2019. 321: 929–937.

[30] Berezin M.Y. Achilefu S. . “Fluorescence Lifetime Measurements and Biological Imaging”. Chem Rev. 2010. 110(5): 2641–2684. 20356094 10.1021/cr900343z PMC2924670

[31] Glanzmann T. Ballini J.-P. van den Bergh H. Wagnières G. . “Time-Resolved Spectrofluorometer for Clinical Tissue Characterization During Endoscopy”. Rev. Sci. Instrum. 1999. 70(10): 4067–4077.

[32] Fischbach T. Duschek F. Hausmann A. Pargmann C. , et al. “Standoff Detection and Classification Procedure for Bioorganic Compounds by Hyperspectral Laser-Induced Fluorescence”. Proc. SPIE. (2015). 9455: 945508

[33] Shkolyar S. Lalla E. Konstantindis M. Cote K. , et al. “Detecting Ce3 + as a Biosignature Mimicker Using UV Time-Resolved Laser-Induced Fluorescence and Raman Spectroscopy: Implications for Planetary Missions”. Icarus. 2021. 354: 114093.

[34] Petrich J.W. Chang M.C. McDonald D.B. Fleming G.R. . “On the Origin of Nonexponential Fluorescence Decay in Tryptophan and its Derivatives”. J. Am Chem. Soc. 1983. 105(12): 3824–3832.

[35] Gudgin E. Lopez-Delgado R. Ware W.R. . “The Tryptophan Fluorescence Lifetime Puzzle. A Study of Decay Times in Aqueous Solution as a Function of pH and Buffer Composition”. Can. J. Chem. 1981. 59(7): 1037–1044.

[36] Pesek J.J. Abpikar H. Becker J.F. . “Fluorescence Lifetime Measurements of Mercury/Protein Complexes”. Appl. Spectrosc. 1988. 42(3): 473–477.

[37] McGuinness C.D. Macmillan A.M. Sagoo K. McLoskey D. Birch D.J.S. . “Excitation of Fluorescence Decay Using a 265 nm Pulsed Light-Emitting Diode: Evidence for Aqueous Phenylalanine Rotamers”. Appl. Phys. Lett. 2006. 89(6). 063901

[38] Lopez-Delgado R. Lazare S. . “Fluorescence Properties of Methyl Salicylate in Vapor, Liquid, and Solution”. J. Phys. Chem. 1981. 85(7): 763–768.

[39] Htun T. Klein U.K.A. . “Laser-Induced Fluorescence Decays of Polyethylene Films”. J Lumin. 2010. 130(7): 1275–1279.

[40] Salas Redondo C. Kleine P. Roszeitis K. Achenbach T. , et al. “Interplay of Fluorescence and Phosphorescence in Organic Biluminescent Emitters”. J. Phys. Chem. 2017. 121(27): 14946–14953. 10.1021/acs.jpcc.7b04529 PMC6614881 31303904

[41] Kalytchuk S. Wang Y. Polakova K. Zboril R. . “Carbon Dot Fluorescence-Lifetime-Encoded Anti-Counterfeiting”. ACS Appl. Mater. Interfaces. 2018. 10(35): 29902–29908. 30085654 10.1021/acsami.8b11663

[42] Onuma T. Kudo K. Ono M. Kosaka W. , et al. “Steady-State And Dynamic Characteristics of Deep UV Luminescence in Rock Salt-Structured Mg* _X_ *Zn_1−*X*_O”. J. Appl. Phys. 2023. 134(2): 025102

[43] Wlodarski M. Kaliszewski M. Trafny E.A. Szpakowska M. , et al. “Fast, Reagentless and Reliable Screening of ‘White Powders’ During the Bioterrorism Hoaxes”. Forensic Sci. Int. 2015. 248: 71–77. 10.1016/j.forsciint.2014.12.023 25598484

[44] Lakowicz J.R. . Principles of Fluorescence Spectroscopy. Boston: Springer Science and Business Media, LLC, 2006.

[45] Luchowski R. Gryczynski Z. Sarkar P. Borejdo J. , et al. “Instrument Response Standard in Time-Resolved Fluorescence”. Rev. Sci. Instrum. 2009. 80(3): 033109. 19334909 10.1063/1.3095677

[46] Esposito R. Altucci C. Velotta R. . “Analysis of Simulated Fluorescence Intensities Decays by a New Maximum Entropy Method Algorithm”. J. Fluoresc. 2013. 23(1): 203–211. 23080525 10.1007/s10895-012-1135-0 PMC3556474

[47] Esposito R. Mensitieri G. S G. . de Nicola. “Improved Maximum Entropy Method for the Analysis of Fluorescence Spectroscopy Data: Evaluating Zero-Time Shift and Assessing its Effect on the Determination of Fluorescence Lifetimes”. Analyst. 2015. 140(24): 8138–8147. 26541293 10.1039/c5an01811k

[48] Feuk H. Nilsson S. Richter M. . “Temperature Resolved Decay Time Components of Mg_4_FGeO_6_:Mn Using the Maximum Entropy Method”. Rev. Sci. Instrum. 2023. 94(3): 034901 37012790 10.1063/5.0141346

[49] Fuhrmann N. Brubach J. Dreizler A. . “Spectral Decomposition of Phosphorescence Decays”. Rev. Sci. Instrum. 2013. 84(11): 114902 24289426 10.1063/1.4828353

[50] Zhou P. Hoffmann M.R. Han K. He G. . “New Insights into the Dual Fluorescence of Methyl Salicylate: Effects of Intermolecular Hydrogen Bonding and Solvation”. J. Phys. Chem. B. 2015. 119(6): 2125–2131. 24678946 10.1021/jp501881j

[51] Louis T.A. Ripamonti G. Lacaita A. . “Photoluminescence Lifetime Microscope Spectrometer Based on Time-Correlated Single-Photon Counting with an Avalanche Diode Detector”. Rev. Sci. Instrum. 1990. 61(1): 11–22.

[52] Buchner A. Hadrath S. Burkard R. Kolb F.M. , et al. “Analytical Evaluation of Signal-to-Noise Ratios for Avalanche- and Single-Photon Avalanche Diodes”. Sensors. 2021. 21(8): 2887. 33924194 10.3390/s21082887 PMC8074602

[53] Lawrence W.G. Varadi G. Entine G. Podniesinski E. Wallace P.K. . “A Comparison of Avalanche Photodiode and Photomultiplier Tube Detectors for Flow Cytometry”. Proc. SPIE. 2008. 6859: 68590M

[54] Zhou X. Bec J. Yankelevich D. Marcu L. . “Multispectral Fluorescence Lifetime Imaging Device with a Silicon Avalanche Photodetector”. Opt. Express. 2021. 29(13): 20105. 34266107 10.1364/OE.425632 PMC8237936

[55] Wu J. Liu X. Wang L. Dong L. Pu Q. . “An Economical Fluorescence Detector for Lab-on-a-Chip Devices with a Light Emitting Photodiode and a Low-Cost Avalanche Photodiode”. Analyst. 2012. 137(2): 519–525. 22129542 10.1039/c1an15867h

[56] Atoneche F. Kastberg A. . “Simplified Approach for Quantitative Calculations of Optical Pumping”. Eur. J. Phys. 2017. 38(4): 045703.

[57] Happer W. Jau Y. Walker T. . Optically Pumped Atoms. Weinheim, Germany: Wiley-VCH Verlag, 2010.

[58] Moyon N.S. Singh T.S. Mitra S. . “Fluorescence Studies on the Photophysical Properties and Encapsulation Behavior of Acetaminophen in Different Environments”. Biophys. Chem. 2008. 138(1–2): 55–62. 18819744 10.1016/j.bpc.2008.09.004

[59] Grinberg N. Rodriguez S. . “Ewing’s Analytical Instrumentation Handbook, Fourth Edition”. In: N. Grinberg, S. Rodriguez, editors. Boca Raton, Florida: CRC Press; Taylor and Francis Group, 2019.

[60] Žukauskas A. Vitta P. Kurilčik N. Juršėnas S. Bakienė E. . “Characterization of Biological Materials by Frequency-Domain Fluorescence Lifetime Measurements Using Ultraviolet Light-Emitting Diodes”. Opt. Mater. 2008. 30(5): 800–805.

